# An observational study of post cardiac arrest management at a uk tertiary intensive care unit between 2010 and 2012

**DOI:** 10.1186/2197-425X-3-S1-A199

**Published:** 2015-10-01

**Authors:** A Mahal, A Curtis, R Gray

**Affiliations:** Brighton and Sussex Medical School, Brighton, United Kingdom; Brighton and Sussex University Hospitals Trust, Intensive Care Unit, Brighton, United Kingdom

## Introduction

Patients surviving cardiac arrest account for 5.8% of all UK ICU admissions^1^. Analysis of the Intensive Care National Audit and Research Centre Case Mix Programme Database (ICNARC CMPD) found that 42.9% of patients admitted after cardiac arrest survived to ICU discharge, and 28.6% survived to hospital discharge. Of these 79.9% returned directly home^1^.

## Objectives

To see if our outcomes were comparable to published data and to audit care against the UK Intensive Care Society (ICS) guidelines^2^.

## Methods

Data was collected from our ICU electronic information system, including cardiac arrest in or out of hospital (IHCA/OHCA) and initial cardiac rhythm, either Ventricular Fibrillation/Tachycardia (VF/VT) or Asystole/Pulseless Electrical Activity (PEA). We recorded implementation of the ICS Post Cardiac Arrest Care Bundle, comprising; coronary reperfusion, haemodynamic optimisation, control of ventilation, blood glucose, temperature and seizures. Hospital survival and discharge data were collected from the electronic hospital discharge database.

## Results

161 patients were admitted post cardiac arrest between January 2010 and December 2012. 6 patients were excluded due to incomplete data. There were 60 IHCA, 65% male (mean age, 68 years) and 35% female (mean age, 61 years) and 95 OHCA, 69% male (mean age, 66 years) and 31% female (mean age, 70 years). Comorbidities included cardiovascular (36%), gastrointestinal (20%) and drug/alcohol abuse (17%).

The predominant rhythm was VF/VT (56%) in OHCA and Asystole/PEA (65%) in IHCA. 98% of patients were mechanically ventilated and 79% required haemodynamic optimisation with inotropic agents. Adequate blood glucose control was achieved in 72% of patients. Primary PCI was performed in 20% VF/VT OHCA. Cooling was achieved in 78% of VF/VT OHCA compared to 24% of VF/VT IHCA.

58% of the cohort died during hospital admission, 86% during their ICU stay. However 42% went on to survive to hospital discharge, 78% of whom were discharged home (33% of all patients admitted). The best outcome was seen in VF/VT IHCA and VF/VT OHCA; 62% and 50% respectively survived to hospital discharge. In VF/VT IHCA 77% of hospital survivors were discharged home compared to 81% in VF/VT OHCA. Poorest outcome was observed in PEA/Asystole OHCA where 27% survived to hospital discharge.

## Conclusions

Comparing our population to the ICNARC CMPD data, survival to hospital discharge (33% vs. 28.6%) and subsequent discharge home (78% vs. 79.9%) were comparable. Adherence to the ICS bundle of care was well achieved overall. The poorest component of the bundle was provision of primary PCI for OHCA VF/VT.
